# Voltage-clamp fluorometry for advancing mechanistic understanding of ion channel mechanisms with a focus on acid-sensing ion channels

**DOI:** 10.1042/BST20240165

**Published:** 2024-10-14

**Authors:** Eleonora Centonze, Stephan Kellenberger

**Affiliations:** Department of Biomedical Sciences, University of Lausanne, 1011 Lausanne, Switzerland

**Keywords:** acid-sensing ion channels, conformational changes, fluorescence measurements, ion channels, voltage-clamp fluorometry

## Abstract

Voltage-clamp fluorometry (VCF) has revolutionized the study of ion channels by combining electrophysiology with fluorescence spectroscopy. VCF allows ion channel researchers to link dynamic structural changes, measured in real time, to function. Acid-sensing ion channels (ASICs) are Na^+^-permeable non-voltage-gated ion channels of the central and peripheral nervous system. They function as pH sensors, triggering neuronal excitation when pH decreases. Animal studies have shown the importance of ASICs for pain and fear sensation, learning, and neurodegeneration following ischaemic stroke. This review explores the technical bases and various developments of VCF, including fluorescence resonance energy transfer and the use of unnatural fluorescent amino acids. We provide an overview of VCF applications with a focus on ASICs, detailing how VCF has unveiled proton-induced conformational changes in key regions such as the acid pocket, wrist, and pore, crucial for understanding transitions between closed, open, and desensitized states.

## Introduction

Ion channels are transmembrane proteins that allow the movement of ions across membranes. They are essential for fast electrical signalling in excitable tissues, controlling numerous physiological processes such as neuronal signalling, muscle contraction, and hormone secretion [1]. Essential for their function is the ability to undergo conformational rearrangements to allow the passage of ions along the electrochemical gradient in response to various stimuli, as ligand binding, voltage changes, mechanical forces and temperature changes [2]. In most ion channels, the channel domains controlling activity, e.g. the voltage sensors or modulator/ligand binding sites, are distant from the channel gates, which are typically located in the channel pore. A change in membrane potential or binding of a ligand can induce rearrangements of the three-dimensional structure that may eventually lead to the opening of the gate(s). Understanding the mechanisms of signalling between the ligand-binding sites or voltage sensors and the gates is of fundamental interest and may identify target sites for potential pharmacological ligands. To correlate conformational alterations with channel function, classical electrophysiological methods have been combined with optical techniques [3,4]. Voltage-clamp fluorometry (VCF) records simultaneously conformational changes and channel function. In this review, we present the VCF technique, discuss current challenges and developments, and illustrate the use of VCF with the example of acid-sensing ion channels (ASICs).

## Acid-sensing ion channels

**Figure 1.**
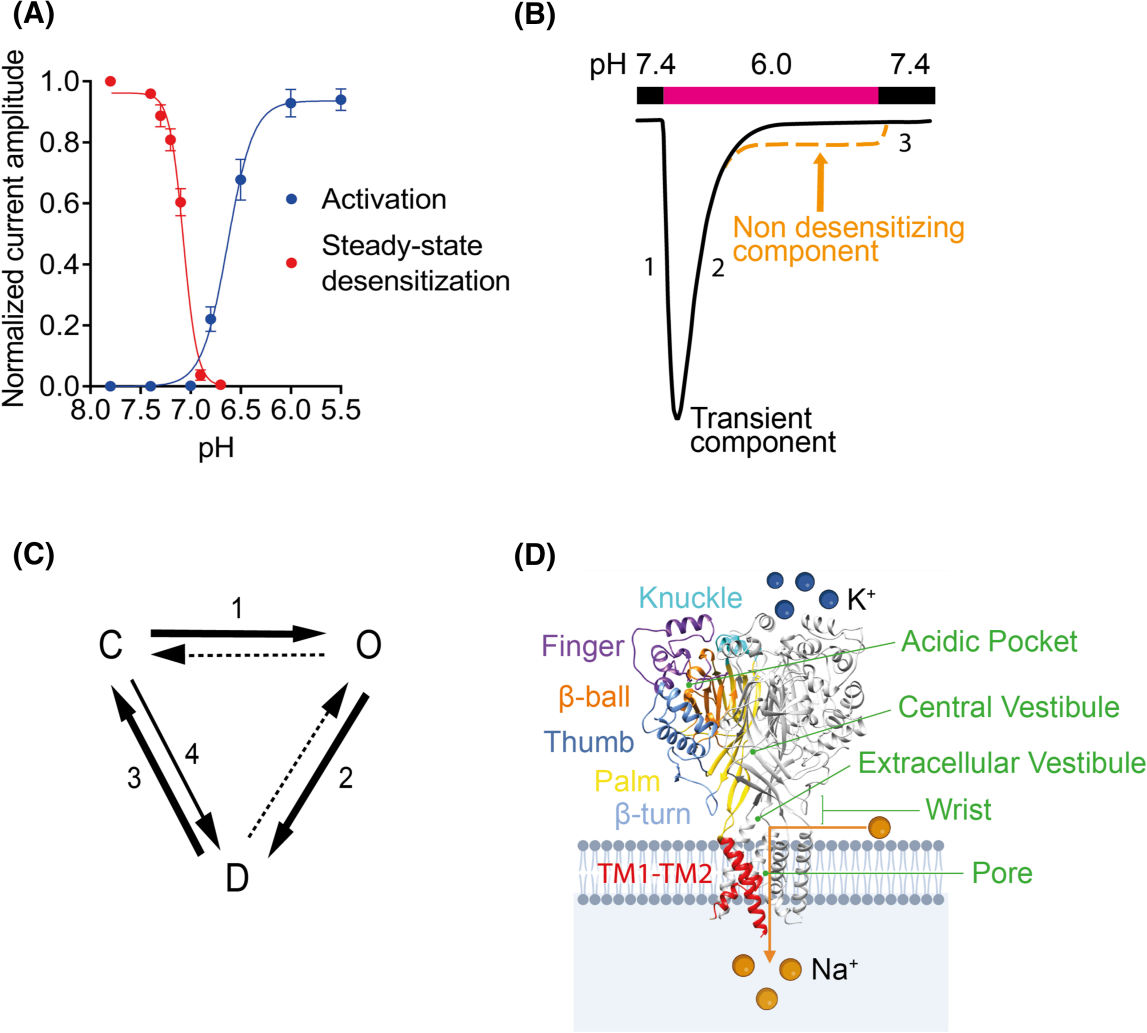
ASIC structure and function. (**A**) pH dependence of activation and steady-state desensitization (SSD), shown for ASIC1a. For SSD, the cells were exposed to the indicated conditioning pH during 60 s, before measurement of the fraction of non-desensitized channels by an acidification to pH 6.0. For activation, the normalized current amplitude is represented as a function of the stimulating pH. (**B**) Representative ASIC current trace measured at −60 mV from *Xenopous* oocytes expressing ASIC1a. The extracellular pH was changed from pH 7.4 to 6 as shown by the horizontal bar. The numbers indicate different phases of the current, corresponding to transitions shown in C. The dashed orange line illustrates an ASIC current with incomplete desensitization. (**C**) Gating model describing ASIC activity. Numbers in B and C indicate transitions, such as 1 = opening, 2 = desensitization, 3 = recovery from desensitization, and 4 = SSD. (**D**) Structure of ASIC1a trimer, based on the crystal structure obtained from chicken ASIC1 [26]. In one ASIC subunit, five distinct domains are highlighted by different colours, as indicated [7]. The location of different vestibules and the pore is indicated with green text.

ASICs belong to the degenerin/epithelial sodium channel family (Deg/ENaC), a group of voltage-insensitive cation channels expressed in the nervous system and epithelial cells [5]. ASICs are proton-gated, Na^+^-permeable ion channels. The subunits ASIC1a, ASIC1b, ASIC2a, ASIC2b, and ASIC3 form trimeric, functional channels by the assembly of homologous or identical subunits [6,7]. The pH dependence of ASIC activation can be determined by measuring the current response under various pH conditions, yielding a pH of half-maximal activation, pH_50_, of ∼6.5 for ASIC1a and ASIC3 [6,8,9] (Figure 1A). The ASIC current is transient, because shortly after opening, the channel enters a non-conductive desensitized state in the continuous presence of the low pH (Figure 1B,C). A weakly acidic pH causes steady-state desensitization (SSD), where the ASIC transitions from the closed to the desensitized state without apparent opening, and becomes unresponsive to further extracellular acidification [10]. Channel properties such as ion selectivity, pH-dependence, and kinetics depend on the subunit composition [11]. ASIC1a homomeric channels are also permeable to Ca^2+^ ions, suggesting a possible role in calcium signalling pathways [12,13].

The ASICs show the highest expression in the nervous system. The activation of ASICs in neurons induces a neuronal depolarization and thus generally a neuronal excitation [13]. Animal studies have shown that ASICs are involved in pain perception, especially in acute and inflammatory pain [14–16]. ASICs have been associated with neurological conditions such as migraine [17], neurodegenerative diseases [18], epilepsy [19], neuronal death after ischaemic stroke [20], and psychiatric disorders such as anxiety and depression [21].

ASIC subunits feature two transmembrane domains with intracellular N- and C-termini, alongside a large extracellular loop. Atomic scale structures of chicken [7] and human [22,23] ASIC1 described the subunit as resembling a hand holding a ball with defined domains named wrist, palm, finger, knuckle, thumb and β-ball (Figure 1D). The acidic pocket (AcP), comprised of a β-ball surrounded by finger, thumb, knuckle and palm domains, contains many acidic residues, and is oriented towards the channel's outer surface, sufficiently exposed to bind ligands such as peptide toxins [22,24–26]. The extracellular vestibule, located at the entrance of the pore at the level of the wrist, the central vestibule positioned above it, and the three AcPs (Figure 1D) contain high densities of acidic residues and constitute binding sites for modulators and drugs [24–29]. The palm connects directly to the transmembrane helices, TM1 and TM2, via the β1 and β12 strands, respectively, and to the thumb domain via the β9 and β10 strands. It spans almost the entire height of the extracellular domain. The two transmembrane helices cross the lipid bilayer, forming the channel gate in the upper third and the selectivity filter in approximately the centre of the membrane [24,30–32]. The pore is formed by the TM2 helices. Each TM2 segment is separated into two parts at the level of the selectivity filter where a horizontal, extended loop connects the upper and lower part, in a way that the lower TM2 of one subunit continues the upper TM2 helix of a neighbouring subunit. This region plays a key role in ion selectivity of ASICs [7].

The acid pocket, wrist and palm contain titratable residues whose mutation affects the pH dependence, and which may thereby act as H^+^ sensors for activation and/or desensitization [33–38]. Extracellular acidification induces protonation of such residues, triggering a cascade of structural rearrangements in the channel protein, ultimately resulting in the opening of the pore. Conducted ions reach the pore from the extracellular side through lateral fenestrations at the level of the wrist (Figure 1D). Despite the progress in recent years, many open questions regarding ASIC activation remain.

## Principles of VCF

### Historical perspective

Cryo-electron microscopy and crystallography offer detailed structural insights into ion channels, but they lack dynamic information. VCF combines the measurement of ionic currents and fluorescence intensity of environmentally sensitive fluorophores that have been introduced at sites of interest. VCF was developed by the Isacoff and the Bezanilla laboratories to detect structural changes in ion channels and associate them with functional transitions [3,4]. VCF indicates (1) whether a conformational change occurs in the proximity of the introduced fluorophore and (2) when this change happens.

**Figure 2.**
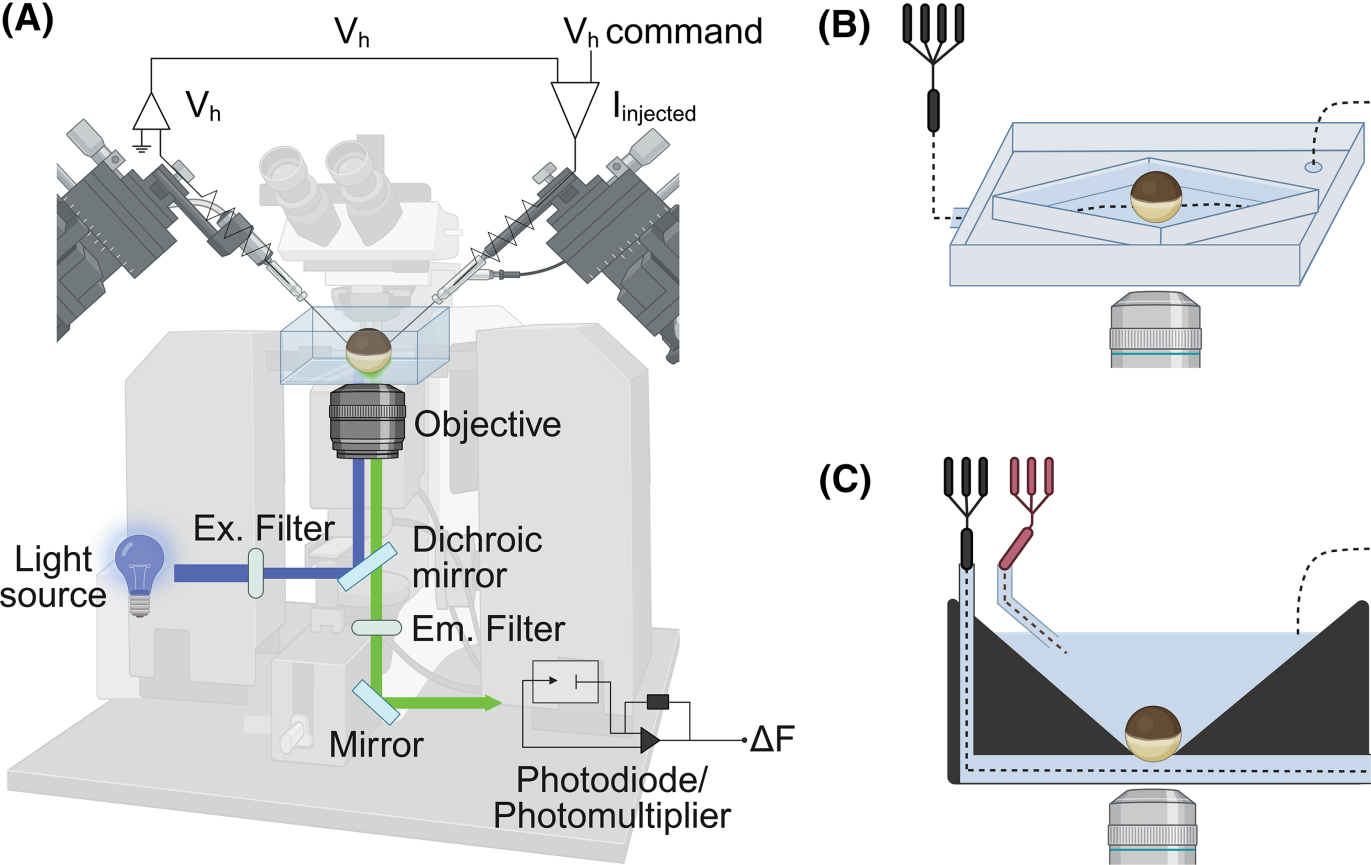
VCF recording setup. (**A**) Schematic illustration of a recording VCF setup. A light source illuminates the oocyte by passing through a filter, a dichroic mirror, and the objective, to excite the fluorophores conjugated to proteins expressed on the surface of the oocyte. Stimulated residues emit a fluorescent signal that is filtered and transmitted to the detection system such as photodiode or photomultiplier. Top, the main components of a TEVC. The potential difference between the bath and the potential electrode *V_h_*, is compared with the command potential, *V*_comm_ and determines the amount of current injected into the oocyte through the current electrode. A dissecting microscope is used to visualize the chamber for placing the oocyte and the electrodes. (**B** and **C**) Schematic view of oocyte chambers used for the measurement of currents and fluorescence signals (Δ*F*) showing (**B**) open diamond bath recording chamber, in which the oocyte is completely perfused by the extracellular solution and (**C**) two-compartment recording chamber with the oocyte sitting in a hole, exposed to two compartments. The agonist is applied via the lower compartment.

The first VCF study from the Isacoff laboratory measured conformational changes of voltage-gated Shaker K^+^ channels by coupling epifluorescence measurements with two-electrode voltage-clamp (TEVC) on *Xenopus* oocytes [3], by using a setup equipped to measure simultaneous changes in fluorescence from the surface of the oocyte (Figure 2A). Fluorophores were positioned at the extracellular end of the voltage-sensitive fourth transmembrane segment (S4) of *Shaker* K^+^ channels. Fluorescence signals were correlated to functional changes, providing the first spectroscopic evidence of the movement of S4 during voltage gating [3]. Shortly thereafter, Bezanilla's laboratory adapted the VCF method to the voltage-clamp cut-open vaseline gap configuration. Two detectors were used for fluorescence: a photodiode for detecting changes in fluorescence intensity and a spectrograph for collecting fluorescence emission spectra [4]. Beyond measuring the outward movement of the S4 segment during depolarization, consolidating its role as the main voltage sensor in various channels [39–41], VCF has revealed complex patterns of movement of the S4 segments, which tightly control channel activity. VCF helped to reveal the sequential activation of voltage-gated sodium channels subunits, tracking movement in distinct domains [42,43]. Moreover, this technique was also used to study conformational transitions at the muscle nicotinic acetylcholine receptor [44]. Additional investigations have extended VCF insights to modulation phenomena, uncovering how magnesium binding influences gating dynamics in EAG K^+^ channels by slowing the motion of the voltage-sensing segment S4 [45]. VCF has revealed distinct agonist actions on receptors such as GABA_A_ and adrenoceptor GPCRs [46,47]. Although GPCRs are not ion channels, fluorescence spectroscopy can be applied to them by monitoring fluorescence intensity changes that reflect receptor conformational changes upon ligand binding [46].

In studies involving voltage- and ligand-gated ion channels, discrepancies were observed between the voltage- or ligand concentration-dependence of the change in fluorescence (Δ*F*) and current signals. In *Shaker* K^+^ channels, the fluorescence signal of S4 has a negatively shifted voltage dependence compared with the current, indicating that the fluorescence change (Δ*F*) is more sensitive to voltage changes than ionic current [3]. This shift indicates that the S4 movement represents one step in the chain of conformational changes leading eventually to channel opening. Analogously, in many ASIC mutants, the pH dependence of the Δ*F* signal is shifted to more alkaline values relative to current activation. Consistent with the hypothesis that several sequential conformational changes precede pore opening in ASICs, the pH dependence shift between the Δ*F* and the current is smaller in positions near the pore compared with more distant sites [36,37].

### Δ*F* signals are caused by changes in the fluorophore environment

The VCF signal originates from interactions between the fluorophore and the local environment. The lipophilicity and the presence of polar groups affect the VCF signal. The introduction of amphiphilic molecules into the membrane can alter the signal by integrating into the lipid bilayer and quenching the fluorescence of the fluorophore. During channel activity-related conformational changes, the fluorophore can move between less polar (lipidic) and more aqueous (solvent) environments, affecting fluorescence properties [48].

Quenching amino acids, such as Trp, Tyr, His and Phe can absorb fluorophore energy, reducing the fluorescence intensity. These amino acids have specific functional groups (indoles, aromatic groups, hydroxyls) that can interact with fluorophores [49]. Several studies have used fluorophore-quencher pairing to determine channel residue positioning relative to each other. For example, in a study of the voltage-gated proton channel Hv1, two His residues were identified as potential quenchers for fluorophores placed at a nearby position [48]. Replacing these His residues with nonquenching residues (such as Ala) or strong quenchers (such as Trp) affected the VCF signal [48]. Vullo et al. [35,36] paired fluorophores and quenching groups to determine whether two residues moved towards or away from each other during functional ASIC1a transitions.

Iodide as a soluble quencher was initially used on *Shaker* K^+^ channels [50]. Iodide can be used to test the solvent exposure of a docked fluorophore. By measuring fluorescence quenching at different membrane potentials, researchers could map the iodide accessibility of specific sites within the *Shaker* K^+^ channel [50]. State-dependent access of iodide to some ASIC1a sites revealed exposure of residues in different conformations [37].

### Technical approaches

The most important properties of fluorescent probes used with VCF include environmental sensitivity, photostability, pH dependence, quantum yield, and spectral range [51]. The fluorophore tetramethyl rhodamine-maleimide was used in many studies because of its high environmental sensitivity and photostability [3]. Alternative fluorophores are fluorescein and Oregon Green [4], of which the former is pH sensitive, while the latter is sensitive to Ca^2+^ concentrations. AlexaFluor 488 is a good fluorophore for the study of ASICs due to its low intrinsic pH dependence.

When VCF is used with channels that are activated by an acidification, as ASICs, it is important to test for each docking position whether the observed Δ*F* signal is not due to an intrinsic pH dependence of the fluorophore. A possible test would be to expose the channels first to pH conditions that desensitize the channels, and only then switch to the acidic test pH. If with this protocol the Δ*F* signal persists, it might be due to an intrinsic pH dependence of the fluorophore [37].

The most widely used approach for site-specific fluorophore positioning involves introducing a Cys residue at the target site for covalent labelling with a sulfhydryl-reactive fluorophore derivative. Successful labelling requires that the fluorophore can access and react with the engineered Cys residue. Residues located intracellularly or in the membrane can therefore not be studied by conventional VCF. If endogenous Cys residues that are not involved in disulfide bonds are solvent-accessible, they are removed, generally by mutation to Ser, to avoid non-specific labelling. Appropriate controls need to be carried out with the WT or Cys-less WT channels, to ensure specific positioning of the fluorophore.

For VCF with ligand-gated ion channels, the design of the measuring chamber determines the speed of the solution exchange on which the activation kinetics may depend. It also defines the surface from which current and fluorescent signals are measured. Typically, the optical unit is placed under the oocyte. Many studies have employed open diamond bath recording chambers, in which oocytes are completely perfused by the extracellular solution (Figure 2B) [37,52–54]. In this constellation, the Δ*F* is detected from the downward-facing side of the oocyte pointed towards the objective and towards the solution inlet, while the current is measured from the channels present on the entire oocyte surface. To better correlate the measurement of current and Δ*F* kinetics, measuring chambers were developed in which the solution flows under the oocyte, and the current and Δ*F* signals are measured from approximately the same surface [35,36,55,56] (Figure 2C). The cut-open oocyte system was designed for faster voltage-clamping. The inner chamber, towards which the oocyte is permeabilized, provides electrical intracellular access, while the middle provides electronic guarding. The upper chamber measures simultaneously Δ*F* and current [4,50,57].

Photodiodes and photomultiplier tubes (PMTs) can detect fluorescence signals in VCF experiments. Photodiodes, convert light into electric current via the photovoltaic principle. When photons hit the semiconductor, electron-hole pairs generate a current proportional to the light intensity, offering a compact and cost-effective option for moderate sensitivity applications. PMTs consists of a photocathode emitting electrons when exposed to light, which are then amplified through dynodes, providing higher sensitivity and a wider spectral range for detecting weaker fluorescence signals compared with photodiodes.

### Limitations of VCF

VCF data interpretation has inherent limitations that need to be considered. (1) The mutation and attachment of the fluorophore may change channel function. (2) VCF does not measure movement direction or distance changes. (3) The substantial size of fluorophores may cause detected movement to deviate slightly from the position of the introduced Cys residue. (4) Changes in Δ*F* may also arise from alterations in side chain orientations without protein structure movement. Regarding (1), the function of the fluorophore-carrying channels needs to be carefully analysed to ensure WT-like properties. Points 2 and 3 have been addressed by newer developments discussed below.

### Developments and extensions of VCF

To overcome the limitation that VCF does not provide information on distances, the high sensitivity of fluorescence resonance energy transfer (FRET) has been employed to measure small distance variations. In FRET, energy is transferred from one excited molecule that has absorbed light (donor), to another, (acceptor), if the distance between them is short. This energy transfer depends mainly on the orientation of the donor with respect to the acceptor and the distance between the two. The efficiency drops with the sixth power of the donor-acceptor distance. This makes FRET an excellent tool for measuring distances of typically 2–8 nm [58,59]. Luminescence resonance energy transfer, a version of FRET using a luminescent lanthanide donor, offers precise distance measurements in ion channels with millisecond donor lifetimes. This enables time-resolved detection with less photobleaching, and better signal-to-noise ratios compared with nanosecond-lifetime donors in FRET [60].

Because standard Cys-labelling techniques only provide access to the extracellular space, inward-facing residues could not be investigated in early studies. In 2000, Zheng and Zagotta [61] overcame these limitations by combining fluorescence measurements with patch-clamp. They developed patch-clamp fluorometry (PCF), which they used to record nucleotide-gated channel activity and fluorescence signals from inside-out patches.

Another approach to place fluorophores at solvent-inaccessible positions of the protein is the use of unnatural fluorescent amino acids. The specific incorporation of unnatural amino acids in a protein by ligating them chemically to a tRNA that recognizes the UAG (amber) stop codon was introduced more than 30 years ago [62] and has been used for many applications. The use of unnatural fluorescent amino acids corrects an additional limitation of Cys-based attachment of fluorophores. The conventional fluorophores attached to engineered Cys residues for VCF are quite big and many contain linkers to the sulfhydryl-reactive group. Consequently, the measured fluorescence signal may not exactly represent the position of the engineered Cys residue. In contrast, the unnatural fluorescent side chain is located at the chosen position. ANAP is such a fluorescent unnatural amino acid that has been used in VCF [63,64].

Researchers introduced ANAP as a non-canonical fluorescent amino acid in the ANAP Cyclen-Cu^2+^ resonance energy transfer method, pioneering transition metal ion FRET. ANAP serves as a FRET donor, while transition metal cations like Cu^2+^ bound to TETAC or di-histidine function as acceptors [65,66,67]. FRET efficiency is quantified by reduced ANAP fluorescence post-Cu^2+^ labelling [67]. Zagotta et al. [68,69] further advanced this approach with the non-natural fluorescent amino acid L-acridonylalanine, enhancing sensitivity for studying protein conformational changes over longer timescales. ANAP was also successfully used with conventional VCF for the study of conformational changes in the cytoplasmic domains of the P2X7 receptor, showing independence of movements of the cytoplasmic ‘ballast’ domain of ATP binding [56].

The analysis of a conformational change should always be based on VCF measurements from multiple fluorophore positions [35–37,70,71].

## Applications of VCF in ASIC research

In 2009, Passero et al. [52] labelled E425 in the pore entry of mouse ASIC1a with a fluorescent probe and showed that Δ*F* kinetics correlated with those of current appearance. At the end of the acidic stimulus, when the pH was changed back to 7.4, the fluorescence amplitude returned slowly to the initial value, indicating a slow conformational change during the desensitized-closed transition [52].

Bonifacio et al. [37] investigated fluorescence signals from several positions in ASIC1a during different functional transitions. This study established a timely order of conformational changes in different ASIC domains, showing that the most rapid movements occurred in the finger, the knuckle, the AcP, a palm-thumb loop, and the wrist, while slower movements occurred in the palm. The comparison of Δ*F* and current kinetics showed that slow conformational changes in the palm correlate with desensitization. During recovery from desensitization, finger residues show the earliest backward movements, followed by some residues in the AcP and the palm. The latest movements upon recovery from desensitization were measured in a palm-thumb linker and in the pore entry [37]. Since an endogenous Trp residue was identified, which quenched the fluorescence of fluorophores placed in the finger, it was possible to conclude that the finger moves outward upon acidification.

Vullo et al. [35] measured several fluorophore-quencher pairs to determine specific movements in the AcP of ASIC1a during activation and desensitization. This work identified only a few transient small amplitude Δ*F* signals correlated with ASIC1a activation. Most Δ*F* signals were slower than the current activation kinetics, suggesting rather a role of the AcP in desensitization. The analysis of the conformational changes of the fluorophore-quencher pairs indicated that the AcP collapses upon acidification [35]. This was an experimental proof of the predicted AcP collapse before the structural confirmation of this hypothesis [7,72].

The Pless laboratory has employed high-sensitivity PCF to study the conformational dynamics of ASICs and other channels [73]. This approach allows up to a 10-fold increase in fluorescence signal amplitudes and 50-fold faster time resolution than previous methods. With the unnatural fluorescent amino acid ANAP placed at position 440 in the ASIC1a pore, significant rearrangements were observed upon extracellular acidification. Intracellular application of tetraethylammonium also induced fluorescence changes, together suggesting a highly mobile geometry of the ASIC1a protein around its central pore [73].

**Figure 3.**
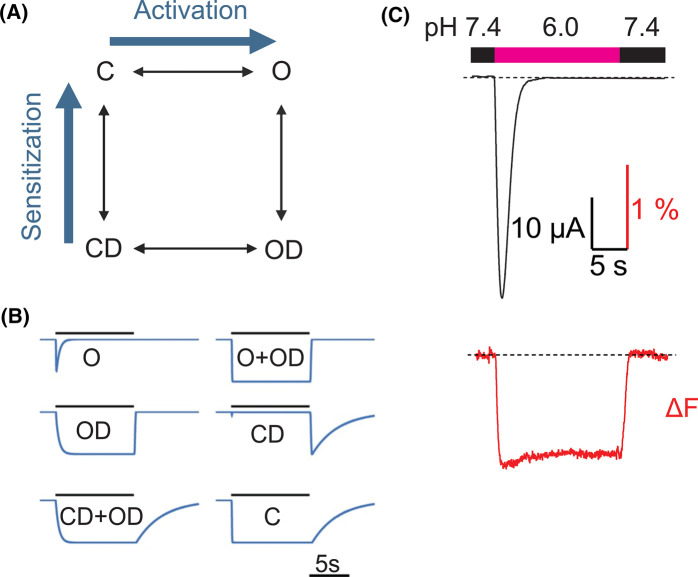
VCF recordings from ASIC1a. (**A**) Kinetic model used for simulation of fluorescence traces; C, closed; O, open; OD, open-desensitized; CD, closed-desensitized. (**B**) Model-generated fluorescence traces. The model is used to generate traces for a pH change from a conditioning pH7.4 to pH6.0. The states or combination of states to which a value of −1 was attributed are indicated with each trace. (**C**) Representative current (black) and Δ*F* (red) hASIC1a traces, recorded simultaneously from an oocyte under voltage clamp to −40 mV. Bars indicate the current and Δ*F* amplitude and the timescale. The timing of the extracellular solution changes is indicated in the bar above the traces.

Vullo et al. placed fluorophores at both distal and proximal sites relative to the pore of hASIC1a and used rapid perfusion to measure Δ*F* and current signals. Rapid movements occurred at sites distal, such as the finger and acid pocket, and near the pore. This indicated that distal sites most likely trigger conformational changes for channel activation in response to extracellular acidification [36]. Using strategic placement of fluorophore-quencher pairs and kinetic analysis of Δ*F* and current signals, this study found rapid conformational changes in the palm-thumb loop containing the β-turn, which interacts with the top end of TM1. In contrast, conformational changes in the palm were slower. Based on these observations it was concluded that conformational changes for channel opening are likely transmitted towards the pore through a pathway involving the β-turn region in the thumb-palm loop and the TM1 rather the ‘internal pathway’ via the palm [36]. To rationalize the interpretation of Δ*F* signal shapes, this study used a kinetic model based on the Hodgkin-Huxley formulation, containing the four states open (O), closed (C), closed-desensitized (CD) and open-desensitized (OD; Figure 3A) [74]. To each state a proportionality factor between −1 and +1 was assigned, representing the contribution of that state to the measured fluorescence signal. The model was able to generate Δ*F* traces when assuming that the fluorescence was associated with one or several functional states (Figure 3B). For each mutant, the proportionality factors were adapted to best fit the experimental Δ*F* trace, providing a rational approach for attributing the structural changes observed (Δ*F*) to functional transitions in ASIC1a [36]. For example, the Δ*F* trace of Figure 3C matches most closely a signal associated with both the O and OD states.

The β11–12 linker located in the lower palm of ASIC1a is critically involved in desensitization [24]. Holm et al. [75] mutated N414 of the β11–12 linker to Lys, resulting in a suppression of desensitization, while accelerating tachyphylaxis, the progressive decrease in response during repeated activations. When fluorophores were placed at different positions of ASIC1a, VCF measurements showed that conformational changes in the ectodomain were largely unaffected by the N414K mutation, even at positions close to the pore. In repeated activations, the current amplitudes decreased strongly with time due to tachyphylaxis, while the amplitude of the Δ*F* signals did not change. This study showed that the ectodomain of the desensitization-deficient N414K mutant undergoes very similar conformational changes as do normally desensitizing ASICs, highlighting the complexity of ASIC gating [75].

## Conclusion

By allowing real-time monitoring of structure changes, VCF offers simultaneous insights into ion channel structure, dynamics, and function. In ASICs, this technique has revealed how protons change the conformation of the AcP, wrist, and pore regions during channel activity.

## Perspectives

- VCF is a powerful tool for real-time monitoring of ion channel dynamics. It links structural changes to functional transitions.
- VCF and related techniques show how ligand binding or voltage sensing at distant sites induce structural changes to open the channel gate(s) for activation.
- Future developments and extensions of VCF will improve the molecular understanding of ion channel function.

## Abbreviations

ASIC: acid-sensing ion channel
FRET: fluorescence resonance energy transfer
OD: open-desensitized
PMT: photomultiplier tube
SSD: steady-state desensitization
TEVC: two-electrode voltage-clamp
VCF: voltage-clamp fluorometry
